# Development and psychometric testing of a questionnaire for assessment of medical science educators’ adherence to ethical principles in virtual education: exploratory sequential mixed methods study

**DOI:** 10.1186/s12909-024-05035-8

**Published:** 2024-01-10

**Authors:** Fateme Mohammadi, Seyed Amin Kouhpayeh, Mostafa Bijani, Shahnaz Karimi, Sanaz Rustaee

**Affiliations:** 1 Chronic Diseases (Home Care) Research Center and Autism Spectrum Disorders Research Center, Department of Nursing, Hamadan University of Medical Sciences, Hamadan, Iran; 2 Department of pharmacology, Fasa University of Medical Sciences, Fasa, Iran; 3 Department of Medical Surgical Nursing, School of Nursing, Fasa University of Medical Sciences, Fasa, 81936-13119 Iran; 4 Department of Medical Education, Medical Education Research Center, School of Nursing, Fasa University of Medical Sciences, Fasa, Iran

**Keywords:** Psychometric Assessment, Questionnaire, Ethics, Virtual education

## Abstract

**Background:**

Principles of ethics are among the pillars of the teaching-learning system. Evaluation of educators’ adherence to principles of ethics in virtual education requires the use of reliable instruments. This study was carried out to develop and test the psychometric properties of a questionnaire for assessment of medical science educators’ adherence to principles of ethics in virtual education.

**Methods:**

This is an exploratory sequential mixed methods study conducted in two parts. In the first stage (the qualitative phase), we used conventional content analysis to establish the concept of ethical principles in virtual education. Thus, 21 semi-structured, in-depth interviews were conducted with 21 medical science professors on a face-to-face basis from March 2022 to November 2022. Subsequently, we developed the items of the questionnaire based on a review of literature and semi-structured in-depth interviews. In the second stage (the quantitative phase), psychometric features of the questionnaire were evaluated using COSMIN criteria (face validity, content validity, construct validity and internal consistency).

**Results:**

Construct validity was surveyed with exploratory and confirmatory factor analysis via completing the questionnaire by 300 medical science professors, who were selected using convenience sampling. The results of exploratory factor analysis yielded a factor loading of the 20 items of the questionnaire to range between 0.79 and 0.98, all the values being significant. The three factors of adherence to the principles of copyright, adherence to educational principles, and justice in evaluation, which were addressed by the instrument, were verified by satisfactory values. The confirmatory factor analysis (CFA) fitted the data well (χ2/df = 13), RMSEA = 0.01, CFI = 0.96, NFI = 0.97, and TLI = 0.99. The total interclass correlation (ICC) of the questionnaire was estimated to be 0.90. Moreover, the reliability of the instrument measured in terms of internal consistency was estimated 0.98.

**Conclusion:**

The findings of the study indicated that the questionnaire we developed for evaluation of adherence to ethical principles of in virtual education was valid and reliable enough. Therefore, the managers in the education system can employ this instrument to assess medical science educators’ adherence to principles of ethics in virtual education.

## Introduction

In most universities across the world today, education has witnessed fundamental changes as a result of advances in technology, and face-to-face education has given way to virtual or blended methods of education [1], a process expedited by the COVID-19 pandemic [2–3].

Virtual learning for professions related to medical sciences must meet the healthcare needs of society and prepare the learners for delivering effective clinical services by equipping them with the required skills [4]. The Internet has eliminated many of the limitations in education and provided countless benefits, including security, personalization, and better services for educators and learners [5]. However, educators are faced with a few issues of concern in online classes. Ambiguities in the principles and rules of ethics in virtual education can have many consequences [6]. One of the essential qualities of a satisfactory educational environment is creating an atmosphere where the learners do not experience tension [7], and one of the professional duties of university teachers toward providing a healthy learning environment is observing the ethical principles [8]. The presence of an ethical framework in education in virtual environments plays a crucial part in shaping the learners’ perception of ethics, as well as their ethical development and professional commitment [9]. Various studies report that not only the educational principles of ethics, but also the ethics of communication should be emphasized in e-leaning environments [10].

In Iran, since 2020, with the onset of COVID-19, learning in all levels of education, from elementary schools to universities, has become online and has mostly remained so despite a significant reduction in COVID-19 cases. In schools and universities today, e-learning is like a newborn which needs attention to grow properly. It is unlikely that the learning environment will ever change completely back to its pre-COVID-19 status.

Differences between learners and educators in different cultures have led to variations in virtual learning environments [10]. For example, in western cultures, individuals are willing to participate actively by enabling their webcams and making visual contact, while in many eastern cultures, Iran included, both the learners and teachers are reluctant to use webcams in virtual classes [11]. Accordingly, learning settings should match the learners’ cultural background. Evidently, the principles of ethics in virtual education must be laid in accordance to the dominant culture and religion of a society [8]. Despite the significance of creating the right context for virtual education, the ethics of e-learning are still ambiguous in many cases and research into this area has been scant [10]. As for the regulations and ethics of virtual education, certain general guidelines have been devised for using the Internet, so that educational institutes can incorporate ethical issues in their curricula for online and blended learning [12]. In their qualitative or blended studies, some researchers have presented ethical charters for online learning. In a blended work of research, Salhab (2021) provided an ethical charter for online learning during the COVID-19 pandemic, which was specifically designed for virtual learning in schools [13]. Thus, there is, to the best of our knowledge, no questionnaires for evaluating adherence to principles of ethics in virtual learning environments in universities, and an instrument which comprehensively assesses the educators’ adherence to ethical principles in virtual education is urgently required.

Instrument development experts agree that the content of a questionnaire must be directly derived from the subjects who are the target of the questionnaire [14]. Accordingly, a qualitative approach seemed essential to investigate the concept of principles of ethics and its dimensions and sub-dimensions. Through determining the concepts and definitions and developing the items, qualitative research helps to develop clinical questionnaires [15]. Since we found no standard instruments to be used for evaluation of medical science educators’ adherence to principles of ethics in virtual education and there is a gap in the theoretical and practical knowledge in this area, the researchers decided to develop and test the psychometric properties of a questionnaire for assessment of medical science educators’ adherence to principles of ethics in virtual education. To conduct the present study, the researchers used a mixed-method approach. The combination of qualitative and quantitative designs helps to have a better understanding of the subject under study, compensates for the defects of either design used separately, enhances the validity and reliability of the study, and creates new boundaries in knowledge [16]. In addition, a mixed-method approach enables the researchers to collect more comprehensive evidence on the research subject and, as a result, detect practical solutions to the research question [17].

Three tools for measuring ethics in education were available to the researchers. The Al-Shehri’s (2017) scale includes 38 questions that are compiled based on the texts. Although the questions of this scale evaluate the principles of ethics in teaching, the psychometric test of the questionnaire was not described. Also, it cannot specifically and comprehensively evaluate the principles of ethics in virtual education [18]. Another scale introduced by Almseidein and KlaifMahasneh (2017) includes 20 questions; the psychometrics of the questionnaire were not described. The researchers did not provide any information on the evaluation of the reliability and validity of their instrument [19]. Another scale prepared by Ayyoub et al. (2022) is designed based on the literature review to measure the level of awareness of electronic crimes pertaining the electronic learning among Jordanian university students and includes 38 questions based on a 2-point Likert scale (yes = 0 and no = 1). Although this questionnaire deals with certain aspects of ethics in virtual education, it does not specifically and comprehensively measure compliance with the principles of ethics in virtual education based on Iranian culture [20].

On the other hand, since the aim of the present study was to develop and test a questionnaire psychometrically for assessment of medical science educators’ adherence to principles of ethics in virtual education, the most appropriate design for conducting the study was a sequential exploratory mixed methods design. This approach allows for theoretical investigation and determining the dimensions of complex and multi-dimensional concepts [21]. It also proves useful when the variables are unknown; since there is a lack of a guiding framework or theory, the researcher decided to transfer his/her findings to various groups or aims to develop a new instrument. Therefore, we developed this questionnaire to assess the observance of ethical principles in virtual education in universities of medical sciences.

## Methods

This is a sequential exploratory study carried out to develop an instrument. In a sequential exploratory project, qualitative data are initially collected and analyzed. Next, literature review is done and based on the review of literature the items are added; then, the pool of items is formed. In the quantitative phase, the psychometrics of the instrument is performed, and the validity and reliability of the instrument are estimated [21].

### Phase one (the qualitative stage)

In the present study the qualitative stage was used to determine the medical science professors, perceptions of the concept of ethical principles in virtual education, to identify relevant concepts, and to develop the items.

In the first phase (the qualitative phase) of the study, data were collected through performing semi-structured personal interviews. Semi-structured interview is the most common and important method of data collection in qualitative studies, which relies on asking questions in a predetermined thematic framework and collects important and key data. Thus, 21 semi-structured, in-depth interviews were conducted with 21 medical science professors on a face-to-face basis from March 2022 to November 2022. The study context was four university of medical sciences located in Fars provinces, Iran. Each interview lasted from 45 to 60 min. The inclusion criteria were willingness to participate in the study and at least one year of teaching experience. The subjects who were not willing to participate for any reason were excluded. In the present study, purposeful sampling was applied. The interviews started with a general question, “What is your teaching experience?”, followed by more specific questions, including: “Can you describe one of your experiences of teaching an online class?”, “Based on your experience, what do principles of ethics mean in virtual education?”, “What ethical issues and challenges have you encountered in virtual education?”, and “Based on your experience, what do principles of ethics in virtual education include?” Also, follow-up questions were asked to increase the clarity of the data provided by the participants. These questions included: “Can you elaborate on the point you just mentioned?”, “What do you mean by that?”, and “Can you share an experience or give an example?”

To analyze the qualitative data, the researchers used Graneheim and Lundman’s approach to content analysis (2004). First, we aimed to immerse in the data and obtain a general understanding, so we read the transcript of each interview several times. Next, the words, sentences, or paragraphs being significant as to the principles of ethics in virtual education were selected as meaning units. Then, we classified the meaning units based on a summary of the meaning of the units and coded the texts. Subsequently, to find the differences and similarities in the codes, we compared them. Then, similar codes were found, and the codes and texts were reviewed. Based on their similarities, the data were classified, so that we can develop the categories. To verify the reliability of the codes, the researchers reexamined the categories and compared them to the data again. The themes were identified after careful and in-depth reflection and comparison of the categories to each other. In general, the following five stages of Graneheim and Lundman’s method of content analysis were followed in the present study:

1. transcribing the whole interview immediately after the completion of each interview.
2. reading the whole transcript of the interview to obtain a general perception of the whole.
3. determining the meaning units and initial codes.
4. classifying the initial codes into larger categories according to their similarities and differences.
5. selecting a title which properly covers the resulting categories [22].

Then, from the qualitative data analysis, a review of the texts was done, and the items were created based on the qualitative study and a review of the texts; then, a pool of items was created.

### Phase two (the quantitative stage/ psychometric properties)

In this study, we used COSMIN (Consensus-based Standards for the Selection of Health Measurement Instruments) criteria [23] to assess the psychometric properties of the questionnaire.

### Psychometric properties (COSMIN criteria)

#### Face validity

To evaluate the qualitative face validity, we interviewed 15 medical science educators face-to-face and asked them to rate the items in terms of difficulty level (difficulty in understanding the words and sentences), relevance (relevance of the items to the dimensions of the questionnaire), and ambiguity (the possibility of misinterpreting the sentences or obscurity in the meaning of the words). After qualitative evaluation of face validity, the faulty items were either revised or eliminated. Moreover, to quantitatively evaluate the face validity and determine the significance of the items, we used the quantitative method of item impact. Thus, 15 experts in instrument development were asked to score each item on a 5-point Likert scale: (5 = Very important; 4 = Important; 3 = Fairly important; 2 = Not very important; and 1 = Not important at all). Finally, all the questionnaires were collected and analyzed. Impact scores higher than 1.5 were regarded as acceptable [24].

#### Content validity

The content validity of the instrument was qualitatively and quantitatively estimated. As to qualitative evaluation of content validity, 15 experts in the fields of instrument development and virtual education were asked to assess the items in terms of necessity, significance, placement, and scoring. To quantitatively evaluate the content validity, the researchers measured content validity ratio (CVR), content validity index (CVI), and scale-level content validity index S-CVI/Ave. A panel of experts was asked to rate CVR on a 3-point Likert scale (Necessary, Useful but not necessary, and Unnecessary). Based on Lawshe’s table, the items whose CVR was over 0.49 can be kept [25–26]. CVI was assessed using Waltz and Bausell’s index. The relevancy, clarity, and simplicity of each of the items were rated on a 4-point Likert scale by fifteen experts in item analysis. Based on the average CVI score of all the items, S-CVI/Ave was estimated. An S-CVI/Ave score of 0.90 or above was considered as acceptable [27–28].

### Item analysis

Prior to exploratory factor analysis, item analysis which is also used to measure the correlation coefficient between the items was employed. In case an item did not have a minimum correlation coefficient of 0.2–0.3 with at least another item, it was eliminated [29].

### Construct validity

#### Exploratory factor analysis (EFA)

Construct validity was measured using factor analysis. The recommended sample size for factor analysis is 5–10 subjects per item [30]. In the present study, the number of selected participants was 15 times that of the items (300 medical science professors). Construct validity using exploratory factor analysis was conducted through Kaiser-Meyer-Olkin index, Bartlett’s test of sphericity, and varimax rotation. After correlation matrix between the variables was calculated, the factors were extracted. The factor loading of each item in the factor matrix and rotation matrix is reported to be at least 0.4 [30]. In the present study, the minimum acceptable degree of correlation between each item and the extracted factors was determined to be a factor loading of 0.5.

#### Confirmatory factor analysis (CFA)

Confirmatory factor analysis was performed using IBM SPSS Amos 21; to determine the usefulness of the model, we used several different indices. Such requirements as a goodness of fit index (GFI) higher than 0.90, root mean square error of approximation (RMSEA) with an acceptance level lower than 0.08, Tucker Lewis Index (TLI) with an acceptance level higher than 0.90, and comparative fit index (CFI) with an acceptance level higher than 0.90 should be met [31].

### Reliability

Internal consistency using Cronbach’s alpha was measured to evaluate the reliability of the questionnaire. A Cronbach’s alpha of 0.7 to 0.8 indicates satisfactory internal consistency [32]. Consistency was estimated using the test-retest method for each factor and the entire scale. Accordingly, the last version of the developed questionnaire was filled out by 100 medical science educators. Two weeks later, the educators were requested to fill out the questionnaire again. The resulting scores of the repeated mesurements were analysed using the test of intraclass correlation coefficient (ICC) [33].

### Ethical considerations

The principles of the revised Declaration of Helsinki, which is a statement of ethical principles that direct physicians and other participants in medical research involving human subjects, were considered in all parts of the present study. All participants signed the informed consent to participate in the study. The participants were assured that all their personal information would remain confidential, and that they were free to withdraw at any stage of the study. We provided them with sufficient information as to the anonymity and confidentiality of their information. Moreover, the Research Ethics Committees of Fasa University of Medical Sciences, Fars, Iran approved the study with the code of IR.FUMS.REC.1400.094.

## Results

In the first phase of the study, 21 unstructured, in-depth, personal interviews with medical science educators were conducted to establish the concept of principles of ethics in virtual education and its dimensions. Analyses of the qualitative data revealed that the principles of ethics in virtual education consisted of the following domains: adherence to the principles of copyright, adherence to educational principles, and justice in evaluation. Initially, there were a total of forty items in the questionnaire. After we reviewed the literature, we added 5 more items, and the questionnaire was designed with 45 items. After holding several meetings by the researchers and a panel of experts, we merged the items that were similar and reduced the number of items to 33. Thus, thirty-three items were used in quantitative analysis. In quantitative evaluation of face validity, ten items had an impact score lower than 1.5; we excluded them and reduced the number of items to 23.

CVR was measured based on the experts’ opnions on the degree of necessity of the items. Based on the Lawshe table, the minimum acceptable value of CVR is 0.33. It was found that the CVR of all the items of the questionnaire ranged from 0.76 to 1; thus, no items were excluded due to unacceptable CVR. The measurement of CVI of each item showed to range between 0.88 and 1. No item was scored below this cut-off point, so all of them were kept. The SCVI/Average was estimated to be 0.99.

**Fig. 1:**
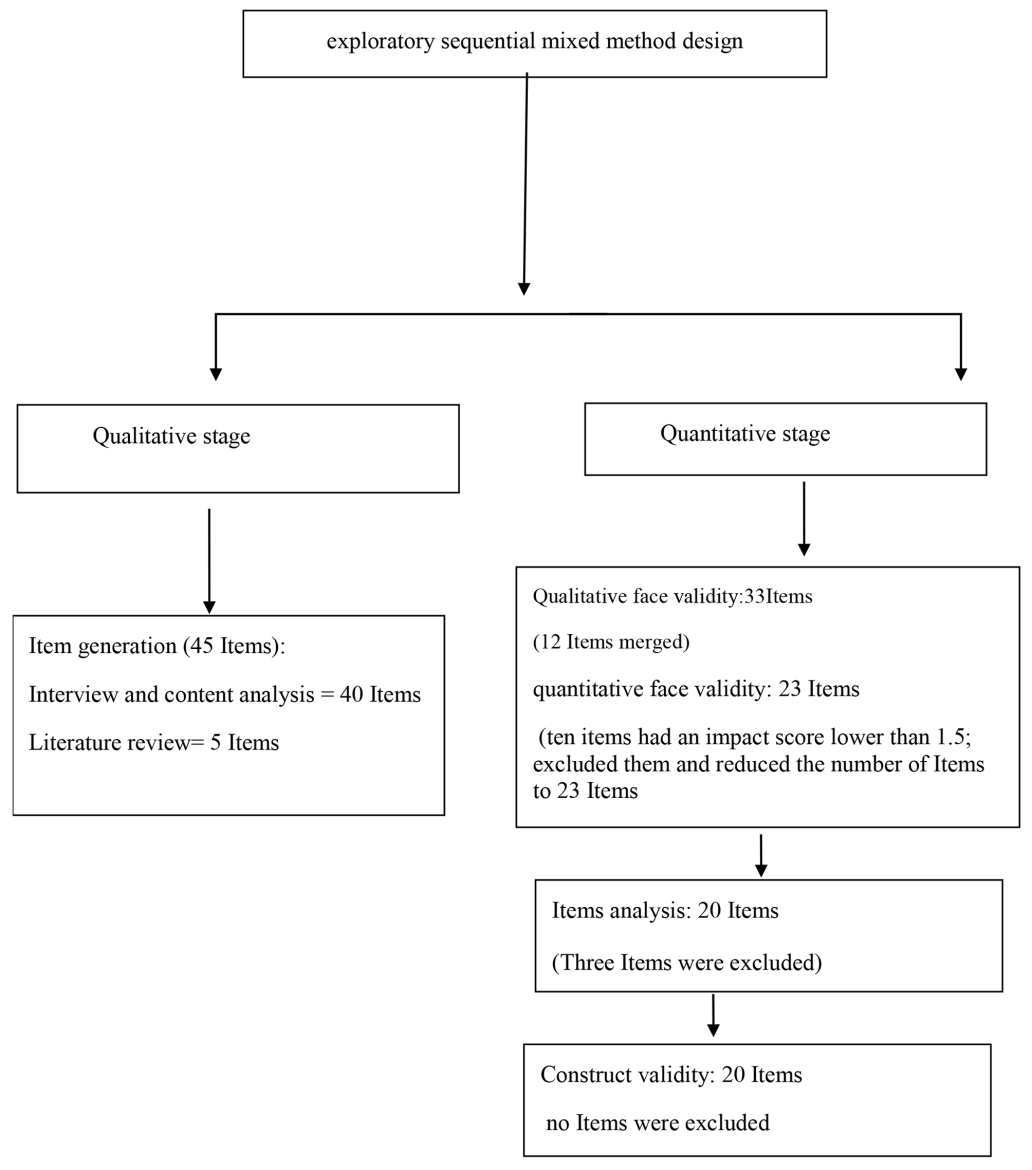
Process of the exploratory sequential mixed method design

Before factor analysis, the items were analyzed by using a sample consisting of one hundred subjects. At this stage, the reliability of the questionnaire, as measured using item analysis, was found to equal a Cronbach alpha of 0.97. The correlation scores of three items were lower than 0.2, so they were eliminated. Finally, 20 items were entered into factor analysis. Figure 1 shows the process of the exploratory sequential mixed method design.

**Fig. 2:**
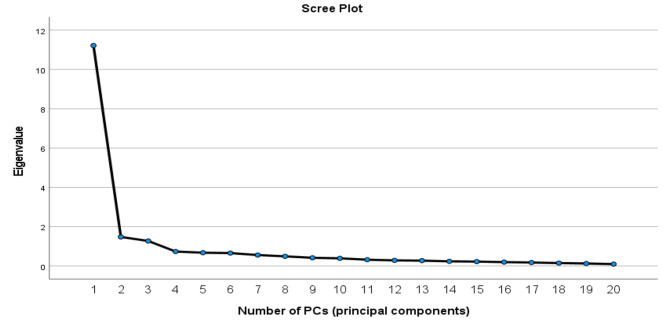
Scree plot to determine number of PCs (principal components)

As to exploratory evaluation of the construct validity of the questionnaire, we selected 15 subjects per item (300 subjects in total). In the first stage of exploratory factor analysis, we used the Meyer-Olkin-Kaiser (KMO) test to determine the adequacy of the sampling, and the result was satisfactory (0.98). Then, Bartlett’s test of sphericity was used to analyze the data. This test shows whether the factor analysis based on the matrix under study is justifiable and appropriate. The results indicated that the chi-square approximation of 4.879 with a degree of freedom of 587 was significant at *P* value of < 0.001. Based on a Scree plot, three factors in the questionnaire were approved. Figure 2, shows the scree plot to determine number of PCs (principal components) that can be extracted.

The results of factor analysis revealed that three factors explained 59.96% of the variance in the adherence to principles of ethics in virtual education questionnaire. In this study, to determine the factors of the questionnaire, the researchers conducted exploratory factor analysis using initial Eigenvalues higher than 1 and a minimum factor loading of 0.4 to keep the items. In this stage, none of the twenty items were eliminated. At the end of this stage, based on the content of the items, the research team identified three factors. The exploratory factor analysis results showed that the factor loading values of the items ranged from 0.79 to 0.98, all of them being significant. Also, the three identified factors were approved by acceptable values. The first factor, adherence to the principles of copyright, consists of six items (items 1 to 6). The second factor, adherence to principles of education, contains nine items (items 7 to 15). The third factor, justice in evaluation, consists of five items (items 16 to 20). Table 1 shows the items and factor loading related to the extracted factors.

**Table 1:** Items and factor loading related to the extracted factors

Factors	Item	Factor loading
Factor 1: Adherence to the principles of copyright	I follow the principles of copyright (I inform my students about the references of the content I present).	0.91
	If I use educational content (PowerPoint slides, books, etc.) created by other individuals, I mention their names in my files.	0.84
	Prior to using educational content created by other individuals, I get permission from the creators.	0.93
	I buy educational content from legal websites.	0.89
	I never present educational content created by other individuals under my own name.	0.80
	I am aware of the principles of ethics in online learning.	0.81
Factor 2: Adherence to educational principles	I conduct my online classes according to the established syllabi.	0.83
	I present educational materials according to a lesson plan.	0.98
	In developing a lesson plan, I consider the topics in the syllabi.	0.80
	I answer my students’ questions in online and offline classes in an effective manner.	0.79
	I give my students access to the educational content from online classes on an offline basis too.	0.82
	I make sure that the educational content I present is of high quality and up to date.	0.81
	I inform my students about the class regulations in online classes.	0.86
	I start my online classes on time.	0.82
	I always treat my students with respect and dignity in my online classes.	0.84
Factor 3: Justice in evaluation	I evaluate my students and correct their homework carefully on a timely basis.	0.96
	In my evaluation of my students in online classes, I consider their active participation.	0.90
	I observe educational justice in grading my students’ work.	0.85
	I carefully check my students’ homework for plagiarism and give appropriate feedback to the offenders.	0.88
	I try to minimize the possibility of cheating on online exams to give everyone equal chances.	0.89

**Fig. 3:**
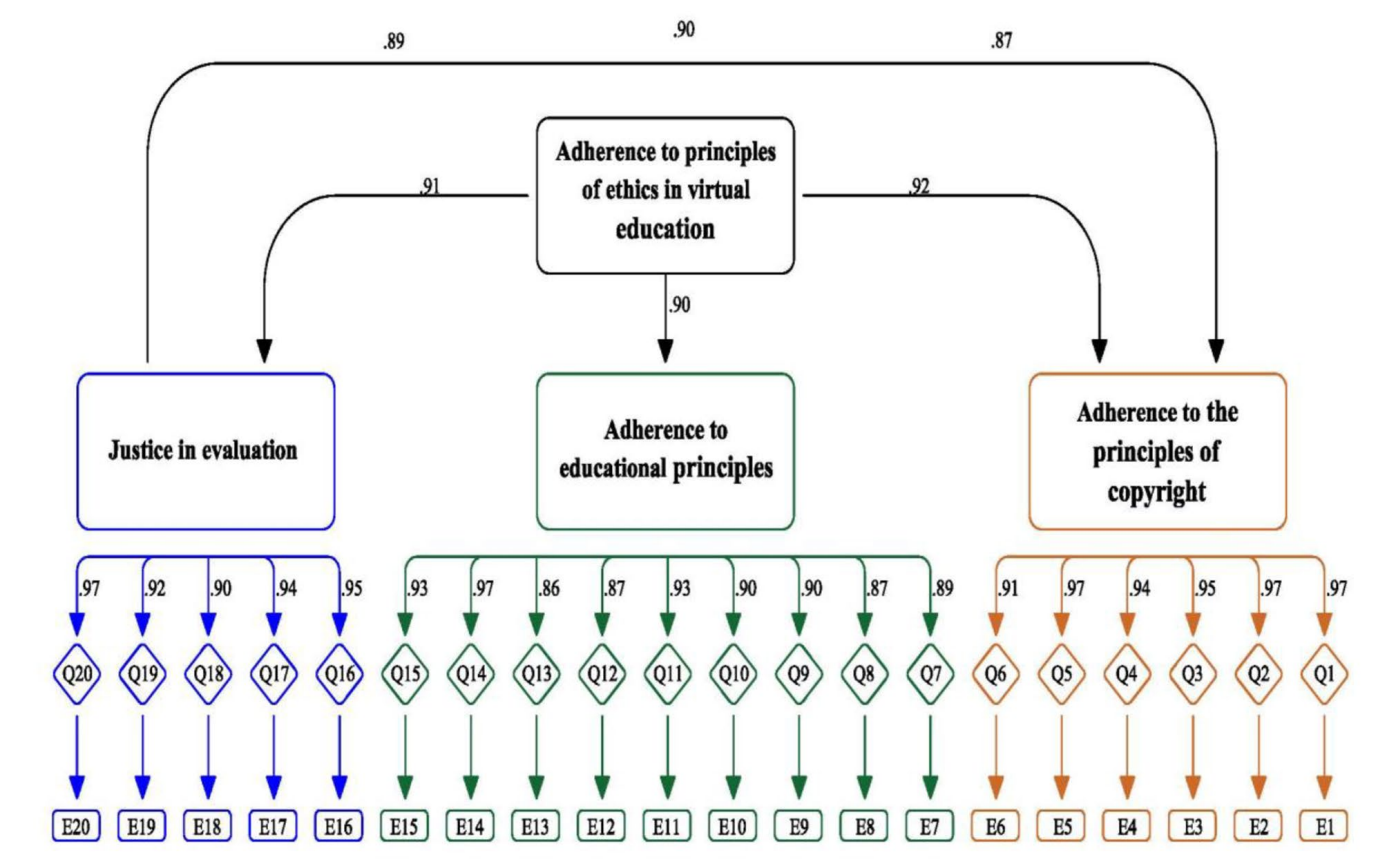
Displays the results of confirmatory factor analysis

The confirmatory factor analysis results pointed to one model with three factors: adherence to the principles of copyright (6items), adherence to the principles of education (9 items), and justice in evaluation (5items). The correlations between the factors and the whole instrument were 0.92, 0.94, and 0.91, respectively. Moreover, a chi-square of 14.49 (df = 13, *P* = 0.001) showed good fitness. The GFI in the present study equaled 0.96, which showed a good fitting. Other indices evaluated in this model were RMSEA = 0.01, CFI = 0.96, NFI = 0.97, and TLI = 0.99, all indicating that the model showed a good fitting. Figure 3 displays the results of confirmatory factor analysis.

To evaluate the reliability of the questionnaire, the researchers measured internal consistency by calculating Cronbach’s alpha. The coefficient was calculated for every factor (subscale) and the entire questionnaire with a sample consisting of three hundred subjects. Cronbach’s alphas higher than 0.7 were regarded as acceptable. An alpha coefficient of 0.89 showed that the present questionnaire had sufficient reliability. Cronbach’s alphas of the subscales of the questionnaire were as follows: adherence to the principles of copyright = 0.94, adherence to educational principles = 0.98, and justice in evaluation = 0.97. Table 2 shows the Cronbach’s alpha of subscales and the entire questionnaire for assessment of medical science educators’ adherence to ethical principles in virtual education.

**Table 2:** Cronbach’s alpha of subscales and the entire questionnaire for assessment of medical science educators’ adherence to ethical principles in virtual education

Factors	Subscale	Items	Cronbach’s alpha
1	Adherence to the principles of copyright	6	0.94
2	Adherence to educational principles	9	0.98
3	Justice in evaluation	5	0. 97
Entire Questionnaire		20	0.98

We measured the consistency of the instrument using the test-retest method. The total intra-class correlation coefficient (ICC) of the questionnaire was 0.90 which was significant at *p* < 0.05. Also, the ICCs of the subscales of the questionnaire were as follows: adherence to the principles of copyright = 0.92, adherence to educational principles = 0.97, and justice in evaluation = 0.89. Table 3, illustrate the intra-class correlation coefficient (ICC) values for the domains of the questionnaire.

**Table 3:** Mean (standard deviation) and intraclass correlation coefficient (ICC) values for the domains of the questionnaire for assessment of medical science educators’ adherence to ethical principles in virtual education

Factor	Dimensions	Mean ± SD	ICC	Confidence interval	*P*-value
1	Adherence to the principles of copyright	23.81 ± 3.93	0.92	0.86–0.93	*p* < 0.05
2	Adherence to educational principles	35.42 ± 6.83	0.97	0.877–0.98	*p* < 0.05
3	Justice in evaluation	20.97 ± 4.28	0.89	0.81–0.96	*p* < 0.05
Entire Questionnaire (Total)		78.20 ± 11.04	0.90	0.83–0.92	*p* < 0.05

### Measurement error

In the present study, by calculating the standard error of measurement and standard error of mean (SEM), we determined the absolute reliability. The results of standard error of measurement for the three subscales were 0.87, 1.32, and 0.52, respectively.

### Repeatability

In addition to stability, the researchers measured agreement which is regarded as positive when the smallest detectable change (SDC) or minimal detectable change (MDC) is higher than the minimal important change (MIC). In the questionnaire developed in this study, the SDCs were higher than the MICs for all the subscales. As to assessment of agreement, the researchers measured SEM. Moreover, we used the split-half technique to assess the internal consistency of the instrument. In the split-half method, we calculated the correlation coefficient between the first half and second half of a questionnaire. As to the present questionnaire, the result was 0.82, indicating a satisfactory reliability.

### Response rate

#### Determination of the ease of use of the questionnaire

To determine the ease of use of the questionnaire, we calculated the average length of time needed for completing the questionnaire and the percentage of individuals who did not respond to all the items. The average time required to complete the questionnaire was estimated to be 6 min, with a range of 5–7 min. Also, an acceptable non-response rate is 0–5%, as shown in the present questionnaire.

Table 4, shows the final version of this self-report questionnaire contains 20 items, which are all scored positively using a 5-point Likert scale: Always = 5 points, Often = 4 points, Sometimes = 3 points, Rarely = 2 points, and Never = 1 point. In this questionnaire, the range of scores is between 20 and 100. The cut-off points of 20–46 = poor, 47–73 = average, and 74–100 = satisfactory determine a respondent’s status.

**Table 4:** The final version of the for questionnaire for assessment of medical science educators’ adherence to ethical principles in virtual education (20 items)

Item	Always	Often	Sometimes	Rarely	Never
1. I follow the principles of copyright (I inform my students about the references of the content I present).					
2. If I use educational content (PowerPoint slides, books, etc.) created by other individuals, I mention their names in my files.					
3. Prior to using educational content created by other individuals, I get permission from the creators.					
4. I buy educational content from legal websites.					
5. I never present educational content created by other individuals under my own name.					
6. I am aware of the principles of ethics in online learning					
7. I conduct my online classes according to the established syllabi					
8. I present educational materials according to a lesson plan.					
9. In developing a lesson plan, I consider the topics in the syllabi.					
10. I answer my students’ questions in online and offline classes in an effective manner.					
11. I give my students access to the educational content from online classes on an offline basis too.					
12. I make sure that the educational content I present is of high quality and up to date.					
13. I inform my students about the class regulations in online classes.					
14. I start my online classes on time.					
15. I always treat my students with respect and dignity in my online classes.					
16. I evaluate my students and correct their homework carefully on a timely basis.					
17. In my evaluation of my students in online classes, I consider their active participation.					
18. I observe educational justice in grading my students’ work.					
19. I carefully check my students’ homework for plagiarism and give appropriate feedback to the offenders.					
20. I try to minimize the possibility of cheating on online exams to give everyone equal chances					

## Discussion

In the present study, we aimed to develop and determine the psychometric features of a questionnaire for assessment of medical science educators’ adherence to principles of ethics in virtual education. Psychometric testing of the questionnaire showed that it had satisfactory face, content, and construct validity and reliability. The extensive review of literature done in this study shows that an instrument which specifically assesses the university teachers’ adherence to principles of ethics in virtual education has not been developed by researchers yet. Accordingly, the authors discuss the findings of the studies which were relatively similar in this field. In 2005 in Chile, Hirsch developed a questionnaire entitled “Attitude scale about professional ethics” to evaluate the university teachers’ perception of professional ethics. Their 55-item scale addresses four competencies related to professionalism: cognitive competence, social competence, ethical competence, and affective-emotional competence. Although this scale evaluates the attitude of teachers involved in higher education toward professional ethics, it does not measure their adherence to principles of ethics in virtual education. Moreover, they exclusively extracted the items of the scale from a review of literature and did not provide any information on the psychometric testing of their instrument [33]. Al-Shehri (2017) used a researcher-made questionnaire consisting of 38 items which were rated on a 5-point Likert scale, ranging from “I completely agree” =5 to “I completely disagree” =1, to assess Code of Ethics of Teaching-Learning in students and teachers. This questionnaire addresses certain aspects of ethics in teaching and learning but does not specifically and comprehensively measure the observance of principles of ethics in virtual education. In addition, the researchers who developed it did not describe the psychometric testing of the questionnaire [18].

In 2017, Almseidein and KlaifMahasneh used a researcher-made questionnaire to measure the learners’ awareness of the ethical aspects of e-learning. The questionnaire consisted of 20 items scored using a 5-point Likert scale, ranging from “I completely agree” =5 to “I completely disagree” =1. However, the researchers did not provide any information on the evaluation of the reliability and validity of their instrument [19]. Ayyoub et al. (2022) employed a researcher-made questionnaire in a study entitled “Awareness of electronic crimes related to E-learning among students at the University of Jordan.” The questionnaire consisted of 38 items scored using a 2-point Likert scale (Yes = 0 and No = 1). Even though this questionnaire addresses certain aspects of ethics in virtual education, it does not specifically and comprehensively measure the observance of principles of ethics in virtual education. Also, the items of the instrument developed by them were developed exclusively based on the results of a review of literature and the psychometric testing of the questionnaire was not described [20]. A review of the available literature showed that although a few studies in different countries have addressed principles of ethics and codes of ethics in teaching and learning in virtual environments, the instruments developed and used by them were not specifically designed for a comprehensive evaluation of university teachers’ adherence to principles of ethics in virtual education. In addition, either the psychometric testing procedures of the instruments were incomplete, or no information was provided on the psychometric testing of the instruments.

### Limitations

The development and psychometric testing of the present questionnaire were conducted in Iran. It is, therefore, suggested that this questionnaire should be translated and tested in other countries. Also, since there were only a few studies on the development and psychometric testing of a questionnaire for assessment of adherence to codes of ethics in virtual learning, the authors discussed the findings of a small number of studies which were relatively close to the subject of the present study.

### Strengths

The present study is the first attempt at developing and evaluating the psychometric properties of a questionnaire measuring educators’ adherence to principles of ethics in virtual education, which is an innovation in its type. Another strength of the study is its complete reliance on a mixed-method approach for the development and psychometric testing of the questionnaire.

## Conclusion

The questionnaire developed in the present study proved to be a reliable and valid instrument for measuring medical science educators’ adherence to principles of ethics in virtual education. The managers in the education system can employ this instrument to assess the educators’ adherence to codes of ethics in virtual education, identify weaknesses, and take the necessary measures to promote the educators’ awareness of professional ethics.

## Data Availability

The data that support the findings of this study are available from the corresponding author upon reasonable request.
